# Utilisation of Reproductive Health Services among Adolescents in Ghana: Analysis of the 2007 and 2017 Ghana Maternal Health Surveys

**DOI:** 10.3390/ijerph21050526

**Published:** 2024-04-24

**Authors:** James Tetteh-Boawolor Ehiawey, Adom Manu, Emefa Modey, Deda Ogum, Edgar Atuhaire, Kwasi Torpey

**Affiliations:** Department of Population Family and Reproductive Health, School of Public Health, University of Ghana, Accra P.O. Box LG 13, Ghana; jtehiawey@st.ug.edu.gh (J.T.-B.E.); eatuhaire@st.ug.edu.gh (E.A.); ktorpey@ug.edu.gh (K.T.)

**Keywords:** adolescent health, sexual health, reproductive health, health service utilisation, Ghana

## Abstract

Early pubertal development induces early sexual activities among adolescents. In Ghana, despite the high sexual activity among Ghanaian adolescents, sexual and reproductive health (SRH) services are underutilised, primarily due to SRH stigma and a lack of SRH knowledge and information. This study examined the use of SRH services among adolescents aged 15–19 years in Ghana over a ten year period. The study utilised data from the 2007 and 2017 Ghana Maternal Health Surveys (GMHSs). Responses from 2056 and 4909 adolescent females captured in the 2007 and 2017 GMHSs, respectively, were used. The results showed a declining utilisation of SRH services among adolescents from 28.3% in 2007 to 22.5% in 2017. The odds of using family planning among sexually active adolescents increased from 2007 [AOR-0.32, CI-(0.135, 0.77), *p* < 0.001] to 2017 [AOR-68.62, CI-(36.104, 130.404), *p* < 0.001]. With increasing age at first sex, adolescents were less likely to use a family planning method in 2007 [AOR-0.94, CI-(0.89,0.99) *p* < 0.001], but this improved in 2017 [AOR-1.26, CI-(1.220, 1.293), *p* < 0.001]. Despite this, knowledge of sources for family planning was found to predict its lower utilisation in both 2007 [AOR = 0.15 (95% CI-0.081, 0.283), *p* < 0.0001] and 2017 [AOR = 0.206 (95% CI-(0.099, 0.426), *p* < 0.001]. The findings show that even though knowledge of family planning methods predicted low utilisation, knowledge of sources, age at first sex, and educational level positively predicted the utilisation of SRH services from 2007 to 2017. Opportunities for both enhancing the clinical environment and health provider attitudes exist and should be explored for improving SRH outcomes among sexually active adolescents in Ghana.

## 1. Introduction

The world is experiencing the largest cohort of adolescents in history [1], with a significant proportion of the global population being between the ages of 10 years and 19 years [2,3]. Despite the awareness that maintaining sexual health in adolescence essentially contributes to reproductive health and well-being in later life [4,5], challenges remain in ensuring access to it. Socio-cultural and gender norms continue to affect both sexes as they navigate their transition to adulthood. Currently, 88% of the 1.2 billion adolescents worldwide live in developing countries where universal access to SRH is yet to be realised and these adolescents face a higher unmet need for SRH services, as well as a higher burden of unplanned pregnancies and contracting sexually transmitted infections (STIs) than their peers in the developed world [3,6].

This may be due to cultural barriers, cost of service, poor provider attitude, and absence of special “adolescent only” services [7,8] that do not encourage pregnant adolescents to access antenatal care [8,9,10,11,12,13,14,15].

Many adolescents engaging in sex for the first time hardly use any form of protection [6,16,17,18] due to casual, impulsive, and unplanned sexual activity among them [19,20]. Adolescents who had an early sexual debut are likely to have multiple partners, thereby increasing their risk of contracting STIs and the risk of unplanned pregnancy [1]. 

The need for adequate attention towards adolescents’ SRH remains critical. Efforts to attain quality SRH are constrained by inadequate access and inequitable distribution of SRH services, resulting in the poor utilisation of SRH services among young people in sub-Saharan African countries. Prior studies show that adolescents across the world face barriers such as long waiting hours, negative provider attitudes, unnecessary restrictions, lack of privacy and confidentiality, social-cultural norms, and stigma when accessing health services [14,21]. There remains a need for relevant data to understand and substantiate the needed interventions [22,23,24]. 

In Ghana, adolescent health is a priority health issue [25,26]. Adolescent- and youth-friendly health services have been identified as a strategy for improving adolescent access to and utilisation of SRH services in the country. Despite its suitability and progress towards improved Sexual and reproductive health and rights (SRHR), outcomes among adolescents are not as expected [27]. The Government of Ghana, through the Ministry of Health and related departments and agencies, has developed several adolescent- and youth-related policy documents and standards, such as the Adolescent Reproductive Health Policy, National HIV/AIDS and STIs Policy, National Health Policy, the Children’s Act (1998), and the Juvenile Justice Act (2003), among others, to support adolescent health. In addition, Ghana ratified several international conventions that promote and protect the well-being of adolescents and youth, such as the United Nations Convention on the Rights of the Child, while the Ghana Health Service (GHS) promotes youth-friendly services. These initiatives, however well-planned, have also not yielded the desired outcome [15] because Ghanaian adolescents still underutilised SRH services, mainly due to stigma around premarital sex [5,28,29,30,31], while over 750,000 adolescents become pregnant annually [31]. 

The challenges of adolescent inaccessibility of SRH services in Ghana have been identified as being due to barriers such as cost of services, lack of awareness about where to obtain contraceptives and STI treatment, misconception about side effects of contraceptives, lack of confidentiality and privacy [32,33], and negative provider attitudes [14,15,26,27,28,29,30,31]. Currently, urbanisation, changes in social norms, and shifting trends in marriage and sexual activity reflect the world in which adolescents are growing [34].

Although there have been many studies on adolescent SRH in Ghana, very few have focused on trends in the utilisation of SRH services among female adolescents nationwide. This study utilises secondary data collected from nationally representative cross-sectional surveys to examine the use of sexual and reproductive health services by adolescents (15–19 years) in Ghana from 2007 to 2017. The findings seek to inform efforts by the government of Ghana to improve and expand access to adolescent SRH services and appropriately respond to common and new barriers to attain optimum SRH.

## 2. Materials and Methods

### 2.1. Study Type

The study was a secondary data analysis of the 2007 and 2017 Ghana Maternal Health Surveys (GMHSs). These were nationally representative surveys among women in the reproductive age of 15–49 years, designed to produce representative estimates for maternal mortality indicators for the country and for each of the three geographical zones, namely Coastal, Middlebelt, and Northern sectors. The Ghana Maternal Health Survey (GMHS) is a household-based survey, which utilises a two-stage sample design. In the 2007 Maternal Health Survey, the first stage involved the selection of samples from a master sampling frame constructed from Enumeration Areas (EAs) from the Ghana Population and Housing Census 2000. The 2017 Maternal Health Survey sampling frame was also based on the Enumeration Areas of the 2010 Ghana Population and Housing Census. The second stage involved the systematic sampling of the households listed from each cluster to ensure an adequate number of completed individual interviews was obtained [35]. The Survey collected data through an interviewer-administered structured questionnaire based on the DHS programme model. Three questionnaires were utilised for the GMHS, as follows: the household, women’s, and verbal autopsy questionnaires. All women aged 15–49 years were eligible to be interviewed from each selected household. For this study, responses from the women’s questionnaire were utilised. 

### 2.2. Data Extraction

For this study, only female adolescents aged 15–19 years were included in the analysis. The Ghana Maternal Health Survey data for 2007 had 10,370 respondents aged 15–49 years, while that of 2017 had 25,062 respondents. Based on the criteria for adolescent females, the number of respondents selected was 2056 for 2007 and 4909 for 2017.

#### 2.2.1. Inclusion Criteria

All female adolescents from the age of 15 to 19 years.

#### 2.2.2. Exclusion Criteria

All female adolescents aged 15–19 years with incomplete responses were excluded from the analysis.

### 2.3. Measures

The outcome variable was the utilisation of SRH services, defined as the use of family planning and abortion services.

**Utilisation of family planning** was a direct Yes/No question “*Are you currently using any method?*” coded as Yes = 1 and No = 0, respectively. 

Family planning methods were classified as modern or traditional methods and recorded. Modern methods include pills, injectables, implants, male condoms, female condoms, intrauterine devices (IUDs), and emergency contraception. Traditional methods also included the withdrawal method, rhythm method, and abstinence.

**Utilisation of abortion services** was measured by the respondents indicating whether they had used safe or unsafe facilities in response to the following question: “*What was the source of the last step to end pregnancy*”. This was recorded as facility type. Based on the criteria set by the Ghana Comprehensive Abortion Care Services Protocol (2012), all hospitals, clinics, and health centres, both public and private, were classified as safe, while private pharmacies, chemical and drug stores, and respondents’ homes were classified as unsafe facilities. Utilisation was thus recorded as a binary variable.

**Provider of the last step to end pregnancy** was recorded as provider type. Providers like doctors, midwives, and nurses are classified as trained; all others, like community health workers, pharmacists, chemical sellers, traditional practitioners, relatives, and friends, were classified as untrained providers. These classifications were all based on the Ghana Comprehensive Abortion Care Standards and Protocols (2012). Independent variables were age, knowledge level, education, sexual activity, and age at first sex.

**Knowledge level** was a composite variable derived from the response (yes or no) to the following four questions: “Have you heard of family planning method”, “Do you know the source of family planning”, “have you ever heard about abortion”, and “do you know where to get abortion”.

**Sexual activity** was binary (yes = 1 or no = 0), following the question: “Have you ever had sex?”. Age at first sex was a follow-up question to sexual activity, by asking the respondent, “What age did you engage in sex?” Responses were captured in single ages.

### 2.4. Data Analysis

Descriptive statistics were used to describe the characteristics of the study participants. This was presented in percentages using frequency tables. Bivariate analysis was carried out using Pearson’s chi-squared test to assess the relationship between independent variables and the utilisation of SRH. Multivariate logistic regression was used to examine the strength of the relationship with SRH utilisation. The odds ratio and the associated 95% confidence intervals were used to assess the strength of the association. A *p*-value of 0.05 was used to determine statistical significance. All statistical analyses were conducted using Stata SE version 15.

## 3. Results

### 3.1. Background Characteristics of Respondents

Table 1 presents the background characteristics of the respondents. The mean age of the respondents was 16.8 years ± 1.4. More than half (52.1%) of respondents in 2007 were from urban areas, while 47.9% were rural dwellers. However, in 2017, more than half (54.1%) of respondents were from rural areas.

In 2007, 90% of the respondents were in-school adolescents, with about three-quarters (75.7%) being in basic school (Primary or Junior High) and 14.7% being educated at Senior High School, with the remaining 10.0% not having formal education. Out-of-school respondents declined from 10% in 2007 to 5.5% in 2017 and respondents who were either in Senior High or Technical School increased to 21.7% from 14.7%. 

Age at first sex was estimated among the respondents. In 2017, the percentage of respondents who first had sex at thirteen years and below was 10.4%, an increase from 9.6% in 2007.

### 3.2. Adolescents’ Knowledge about Sexual and Reproductive Health

In 2007, about 44.7% of adolescents reported that they knew about family planning methods, compared to 59.6% in 2017 [z = 11.41, *p* < 0.001]. With regard to knowledge about the sources of family planning, 46.3% answered in the affirmative in 2007, compared to 60.1% in 2017 [z = 10.05, *p* < 0.001].

With regard to abortion, 89.3% in 2007 and 90.4% in 2017 reported that they knew about abortion [z = 1.50, *p* = 0.066]. About one-third (33.4%) of adolescents in 2007 knew where to access abortion services, this increased to 53.8% in 2017 [z = 14.48, *p* < 0.001]. (Table 2).

### 3.3. Utilisation of Sexual and Reproductive Health Services

In 2007, the proportion of adolescents using a family planning method was 28.3%, which declined to 22.5% in 2017. Approximately 43.1% patronised the services of a trained provider in 2007, whilst 2017 recorded a decline to 35.2%. In 2007, 43.1% of adolescents accessed services in safe facilities compared to 32.8% in 2017 (Table 3).

### 3.4. Background Characteristics and Family Planning

Based on the bivariate analysis of the variables ever had sex and age at first sex both showed a significant relationship with the utilisation of SRH services in 2007 (*p* < 0.05). This relationship included variables: age, highest educational level, and ever had sex in 2017 (*p* < 0.05) (Table 4).

The study also found that knowledge about family planning methods, knowledge of the source of family planning, hearing about abortion, and knowing where to obtain an abortion had significant relationships with the utilisation of family planning methods in 2017; however, none of these variables were found to have a significant relationship with the utilisation of family planning in 2007 (Table 5).

### 3.5. Independent Variables and Abortion Utilisation

The bivariate analysis did not show a significant relationship between the independent variables and abortion utilisation in the 2007 and 2017 studies (Table 6).

### 3.6. Factors Associated with Family Planning Utilisation

In 2007, sexually active adolescents were 68% less likely to use family planning [AOR-0.32, CI-(0.135, 0.77), *p* < 0.001] compared to adolescents who were not sexually active. This changed to 69% in the odds of family planning method use in 2017 [AOR-68.62, CI-(36.104, 130.404), *p* < 0.001]. With increasing age at first sex, adolescents were less likely to use a family planning method in 2007 [AOR-0.94, CI-(0.89, 0.99) *p* < 0.001] and this changed to reflect an increase in the odds of using a method with increasing age at first sex in 2017 [AOR-1.26, CI-(1.220, 1.293), *p* < 0.001]. Additionally, compared to adolescents whose highest level of education was primary level, senior secondary-educated adolescents were more likely to use a family planning method [AOR = 1.822, 95% CI (1.257, 2.643) *p* = 0.002] (Table 7).

Adjusting for the variables of ever hearing of abortion, knowing a source for abortion, and knowledge of the legal status of abortion in Ghana, we found that adolescents who knew about the sources of family planning were still 99.85% less likely to use family planning methods [AOR = 0.15 (95% CI-0.081, 0.283) *p* < 0.001] in 2007 compared to those who did not know about sources of family planning methods. This slightly improves in 2017 [AOR = 0.206 (0.099, 0.426), *p* > 0.001] (Table 8).

## 4. Discussions

The findings of this study show that the utilisation of family planning services remains challenging, as the proportion of adolescents using FP showed a decline from 2007 to 2017, despite the interventions and efforts undertaken by programs in the country. This low utilisation has frequently been reported among adolescents in Ghana [8,36,37,38]. The study also found that over the period under review, adolescents who knew a source of FP were more likely to have utilised FP services less, even though 2017 showed a slight improvement compared to 2007. This finding critically highlights the little progress made in responding to the underutilisation of SRH services among adolescents [38]. Challenges in increasing the utilisation of SRH services remain within the Ghanaian healthcare system and the negative health implications continue to be recorded. It is critical to identify novel opportunities to encourage its utilisation, to respond appropriately to this challenge in Ghana [25,39].

This study revealed that adolescents’ knowledge about family planning services was low in 2007 but had increased in 2017. These findings, though consistent with findings from other SSA countries and multi-country studies [16,22,40], could be a reflection of adolescents’ negative attitudes towards SRH due to cultural barriers, lack of confidentiality at health facilities, fear of side effects, inadequate peer support, and poor provider attitudes as evident from both global and local studies [2,9,19,41]. The lack of comprehensive information on reproductive health issues and services makes adolescent girls vulnerable to unsafe reproductive health behaviour [34,42]. It is plausible that the improvements identified in Ghanaian adolescents in 2017 can be attributed to the interventions towards improved adolescent SRH services [15,25].

This study, however, reported a high knowledge of abortion services among respondents. The proportion of respondents with a knowledge about abortion services in 2007 increased to 90.4% in 2017. A similar pattern was observed concerning the knowledge of a source for abortion services, indicating that adolescents have an increased access to information on this issue [34]. Although this study did not access information sources among adolescents, it is important to explore the sources to assess their validity, since many are unable to seek accurate information from the right source [8,14]. 

Regarding abortion, 43.1% of respondents in 2007 patronised safe facilities, yet, in 2017, it reduced to 32.8%. Similarly, utilisation of the services of a trained professional reduced from 43% in 2007 to 35.2% in 2017, a disturbing trend, suggestive of the increased use of untrained workers. The increase in unsafe abortion, despite the increased awareness and creation of campaigns on safe abortion, could be due to barriers such as cost of services, proximity to services, lack of confidentiality, and privacy [2,26,27,28,29,34,43,44]. Most adolescents feel uncomfortable accessing services of the various components of SRH services in facilities [13,32,40,45]. The multivariate analysis showed a significant association between age at first sex among sexually active adolescents.

The recorded increase in the odds of modern method use from 2007 to 2017 is noteworthy. Many studies identify that the preferred modern method for adolescents is condoms [8,11,36,37,38,46,47,48]. It is plausible that among adolescents who are exposed to information on family planning and contraceptive use, many adolescents opt for the condom because it is cheaper and easily accessible and serves as dual protection [8,10,38], compared to others such as injectables, implants, and intrauterine devices (IUDs). The reduced utilisation of methods that serve pregnancy prevention only roles, but require visitation to health facilities with its associated negative health worker attitudes, inherently acts as a deterrent to adolescents in need [19,28,37].

The multivariate analysis showed a significant association between age at first sex and the utilisation of family planning services. This finding, however, is inconsistent with findings from other studies that revealed that adolescents who initiate sex early are not likely to use family planning methods, which exposes them to the risk of getting pregnant [8,40]. It is plausible that the shift in attitude towards accepting the use of family planning methods is a product of the multiple interventions introduced to improve its utilisation in Ghana [26]. Evidence suggests that older adolescents are likely to be sexually active and, therefore, more likely to utilise SRH services [24,28,29]. With knowledge as a precursor to action, they may have more access to SRH services. 

This study, though nationally representative, is not without its limitations. First, as the study relied on secondary data, this limited our analysis to the variables that had been collected. This study is also cross-sectional and, hence, causal inferences cannot be made. Finally, the study relied on self-reported measures from adolescents, which are subject to recall bias.

## 5. Conclusions

This study identified the factors that are attributable to the decline in the utilisation of SRH services over the period of the 2007 and 2017 Ghana Maternal Health Surveys. The results of this study highlight multiple important issues such as the declining use of family planning and contraceptive methods, amidst the increased knowledge of family planning methods, sources of family planning methods, abortion services, and sources of abortion among adolescents. Our results highlight the important role that socio-cultural concerns play in influencing adolescent attitudes toward the utilisation of SRH services. The underutilisation of these services may occur, even while knowledge of the services is high. 

## Figures and Tables

**Table 1:** Background characteristics of adolescents.

Variables	2007 Frequency (%)	2017 Frequency (%)
Age (Years)		
15	539 (26.2)	1219 (24.8)
16	404 (19.7)	936 (19.1)
17	370 (18.0)	1047 (21.3)
18	411 (20.0)	961 (19.6)
19	332 (16.2)	746 (15.2)
**Highest Educational level**		
No Education	206 (10.0)	271 (5.5)
Primary/JHS	1556 (75.7)	3565 (72.6)
SHS/technical	294 (14.3)	1073 (21.7)
**Residence**		
Rural	984 (47.9)	2658 (54.1)
Urban	1072 (52.1)	2251 (45.9)
**Ever had sex**		
Yes	802 (39.0)	1863 (38.0)
No	1254 (61.0)	3046 (62.0)
**Age at first sex (years)**		
≤13	77 (9.6)	193 (10.4)
14	96 (12.0)	232 (12.5)
15	198 (24.7)	484 (26.0)
16	172 (21.4)	436 (23.4)
17	142 (17.7)	342 (18.4)
18	74 (9.2)	153 (8.2)
19	43 (5.4)	23 (1.2)

**Table 2:** Percentage of adolescents who demonstrated knowledge about SRH.

Variable	2007 Frequency (%)	2017 Frequency (%)
**Heard of Family Planning method**		
Yes	919 (44.7)	2929 (59.7)
No	1137 (55.3)	1980 (40.3)
**Know source of family planning**		
Yes	848 (46.4)	2765 (60.1)
No	978 (53.6)	1839 (39.9)
**Ever heard of abortion.**		
Yes	1780 (89.3)	4320 (90.4)
No	214 (10.7)	458 (9.6)
**Knows where to obtain an abortion**		
Yes	592 (34.4)	2325 (53.8)
No	1132 (65.6)	1995 (46.2)

**Table 3:** Utilisation of SRH by adolescents.

Variable	2007 Frequency (%)	2017 Frequency (%)
**Ever used contraceptives**		
Yes	464 (22.6)	182 (18.4)
No	1587 (77.4)	808 (81.6)
**Currently using any method**		
Yes	227 (28.3)	419 (22.5)
No	575 (71.7)	1444 (77.5)
**Current family planning method**		
Modern method	102 (44.9)	301 (71.8)
Traditional method	125 (55.1)	118 (28.2)
**Type of Abortion Provider**		
Trained provider	25 (43.1)	44 (35.2)
Untrained provider	33 (56.9)	81 (64.8)
**Facility type for last step to end pregnancy**		
Safe facility	25 (43.1)	41 (32.8)
Unsafe facility	33 (56.9)	84 (67.2)

**Table 4:** Background characteristics and utilisation of family planning.

Variables	Utilisation of Family Planning Method					
	2007			2017		
Age	Yes	No	(*p*-Value)	Yes	No	(*p*-Value)
15	27 (73.0))	10 (27.0)	(0148)	19 (1.6)	1190 (98.4)	(0.0001)
16	26 (52.0)	24 (48.0)		35 (3.8)	887 (96.2)	
17	37 (53.6)	32 (46.4)		101 (10.0)	913 (90.3)	
18	67 (49.3)	69 (50.7)		125 (13.5)	777 (86.5)	
19	70 (51.9)	65 (48.1)		139 (20.0)	566 (80.3)	
**Ever attended school**						
Yes	215 (53.)	187 (46.)	(0.594)	399 (8.8)	4115 (91.6)	(0.828)
No	12 (48.0)	13 (52.0)		20 (8.4)	218 (91.6)	
**Highest** **educational level**						
Primary	45 (50.6)	44 (49.4)	(0.509)	59 (6.5)	854 (93.5)	(0.001)
Middle/JHS	109 (46.5)	125 (53.4)		211 (8.3)	2326 (91.7)	
SHS	34 (41.8)	46 (56.2)		128 (16.4)	935 (87.7)	
**Ever had sex**						
Yes	196 (50.4)	193 (49.6)	(0.0001)	409 (24.0)	1287 (76.0)	(0.001)
No	31 (81.6)	7 (18.4)		10 (0.3)	3028 (99.7)	
**Age at first intercourse**						
13 and below	15 (46.9)	17 (53.1)	(0.012)	49 (27.4)	130 (72.6)	(<0.001)
14	23 (52.3)	21 (47.7)		45 (20.8)	171 (79.2)	
15	33 (40.7)	48 (59.3)		105 (23.8)	339 (76.2)	
16	48 (53.3)	42 (46.7)		95 (24.1)	301 (76.0)	
17	42 (55.3)	34 (44.7)		75 (24.3)	234 (75.7)	
18	24 (52.2)	22 (47.8)		32 (23.0)	107 (77.0)	
19	11 (55.0)	9 (45.0)		7 (31.8)	15 (68.2)	

**Table 5:** Knowledge of adolescents about SRH and its utilisation.

Variable	Utilisation of Family Planning Method					
	2007			2017		
	Yes	No	(*p*-Value)	Yes	No	(*p*-Value)
Heard of Family Planning method						
Yes	150 (54.4)	124 (44.9)	(0.278)	304 (10.76)	2521 (89.2)	(<0.001)
No	77 (51.7)	2 (0.72)		103 (3.9)	2541 (96.1)	
Know source of planning						(<0.001)
Yes	140 (97.9)	3 (2.1)				
No	51 (94.4)	3 (5.6)		11. (0.6)	1792 (99.4)	
Ever heard of abortion						
Yes	193 (52.6)	168 (45.8)	(0.320)	351 (8.4)	3835 (91.6)	(<0.001)
No	10 (66.7)	4 (26.7)		12 (2.7)	431 (97.3)	
Knows where to obtain abortion						
Yes	90 (51.4)		(0.320)	244 (10.9)	1997 (89.1)	(<0.001)
No	84 (48.0)	98 (53.3)		104 (5.6)	1751 (94.4)	

**Table 6:** Factors influencing utilisation of abortion by adolescents.

Variables	Facility Type for Last Action					
	2007			2017		
	Safe	Unsafe	(*p*-Value)	Safe	Unsafe	(*p*-Value)
Age						
15	1 (100.0)	0 (0.0)	(0.348)	1 (20.0)	4 (80.0)	(0.700)
16	1 (16.7)	5 (83.3)		4 (26.7)	11 (70.3)	
17	3 (27.3)	8 (72.7)		8 (29.6)	19 (70.4)	
18	4 (16.7)	20 (53.3)		14 (31.1)	31 (68.9)	
19	3 (18.8)	13 (81.3)		14 (42.4)	19 (57.6)	
Ever attended school						
Yes	11 (20.8)	42 (79.3)	(0.968)	40 (34.2)	77 (65.8)	(0.206)
No	4 (80.0)	1 (20.0)		1 (12.5)	7 (87.5)	
Highest Educational level						
Primary	5 (31.3)	11 (68.8)	(0.324)	10 (34.5)	19 (65.5)	(0.996)
Middle/JHS	6 (18.2)	27 (81.8)		22 (33.9)	43 (66.2)	
SHS and Technical	0 (0.0)	4 (100.0)		8 (34.8)	15 (65.2)	

**Table 7:** Association between background characteristics and utilisation of family planning method.

Characteristics	2007				2017			
	COR (95% CI)	*p*-Value	AOR (95% CI)	*p*-Value	COR (95% CI)	*p*-Value	AOR (95% CI)	*p*-Value
Age	0.874 (0.751, 1.017)	0.081	0.94 (0.788, 1.125)	0.511	1.84 (1.694, 1.998)	0.0001	1.20 (1.085, 1.330)	0.0001
Ever had sex	0.23 (0.099, 0.533)	0.0001	0.32 (0.135, 0.765)	0.001	95.7 (50.954, 179.882)	0.0001	68.62 (36.104, 130.404)	0.0001
Age at first sex	0.93 (0.891, 0.975)	0.003	0.94 (0.896, 0.993)	0.000	1.28 (1.242, 1.311)	0.0000	1.26 (1.220, 1.293)	0.0000
Highest Education							Ref	
Primary(ref)	Ref		Ref		Ref			
Junior secondary/JHS	1.173 (0.719, 1.911)	0.522	1.31 (0.781, 2.194)	0.306	1.313 (0.974, 1.771)	0.0746	1.311 (0.944, 1.822)	0.106
Senior Secondary/SHS	1.342 (0.722, 2.495)	0.352	1.54 (0.785, 3.036)	0.208	2.032 (1.472, 2.804)	<0.001	1.822 (1.257, 2.643)	0.002

**Table 8:** Association between knowledge and utilisation of SRH.

Variable	2007		2017	
	AOR (95% CI)	*p*-Value	AOR (95% CI)	*p*-Value
Know the source of FP	0.15 (0.081, 0.283)	0.0001	0.206 (0.099, 0.426)	0.0001
Ever heard of abortion	0.30 (0.170, 0.545)	0.0001		
Knows where to obtain an abortion			1.72 (1.092, 2.701)	0.011
Abortion legal in Ghana	0.93 (0.880, 0.990)	0.023	0.87 (0.472, 1.613)	0.663

## Data Availability

Data were obtained from the DHS program and are available from https://dhsprogram.com/Data/, accessed on 1 December 2023 with the permission of the DHS program.
